# Supplementary figures and images for: An Oncolytic Adenovirus Encoding SA-4-1BBL Adjuvant Fused to HPV-16 E7 Antigen Produces a Specific Antitumor Effect in a Cancer Mouse Model
Source: Vaccines (Basel). 2021 Feb 12;9(2):149. doi: 10.3390/vaccines9020149 (PMC7917608; doi:10.3390/vaccines9020149)

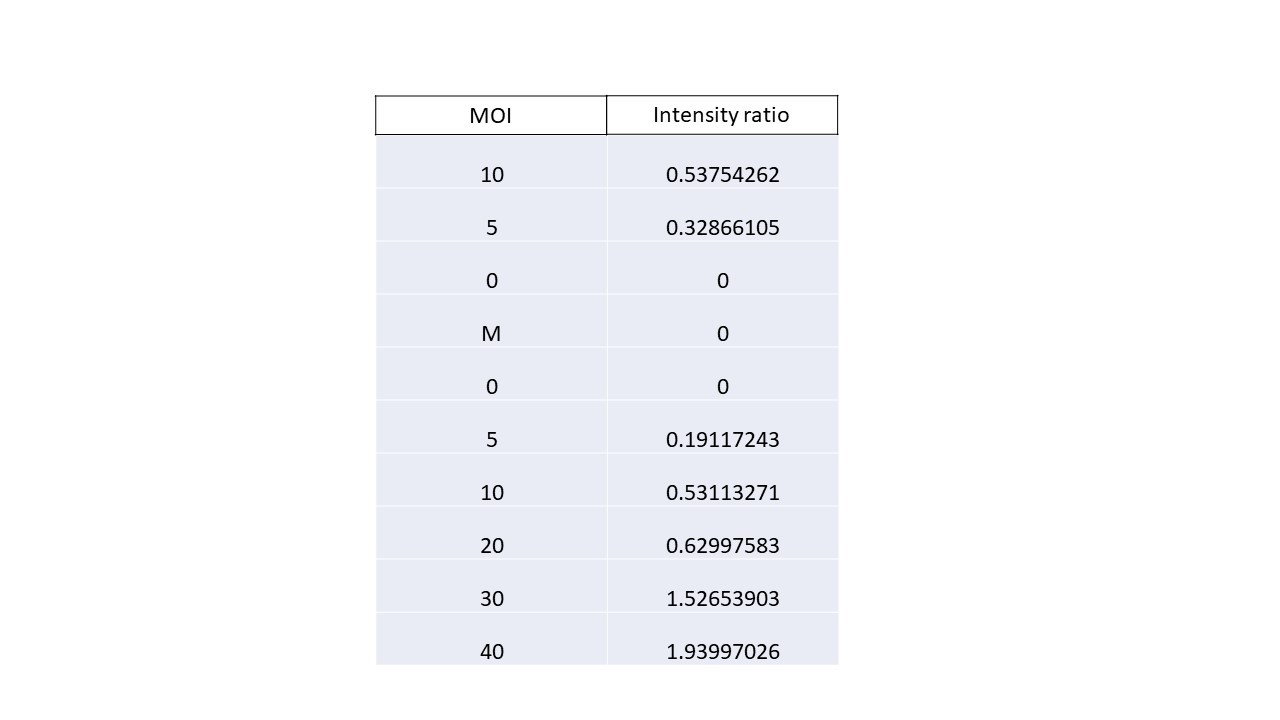

Supplement: Supplementary file 1 [file vaccines-09-00149-s001.zip › Supplementary/Densitometry.jpg]

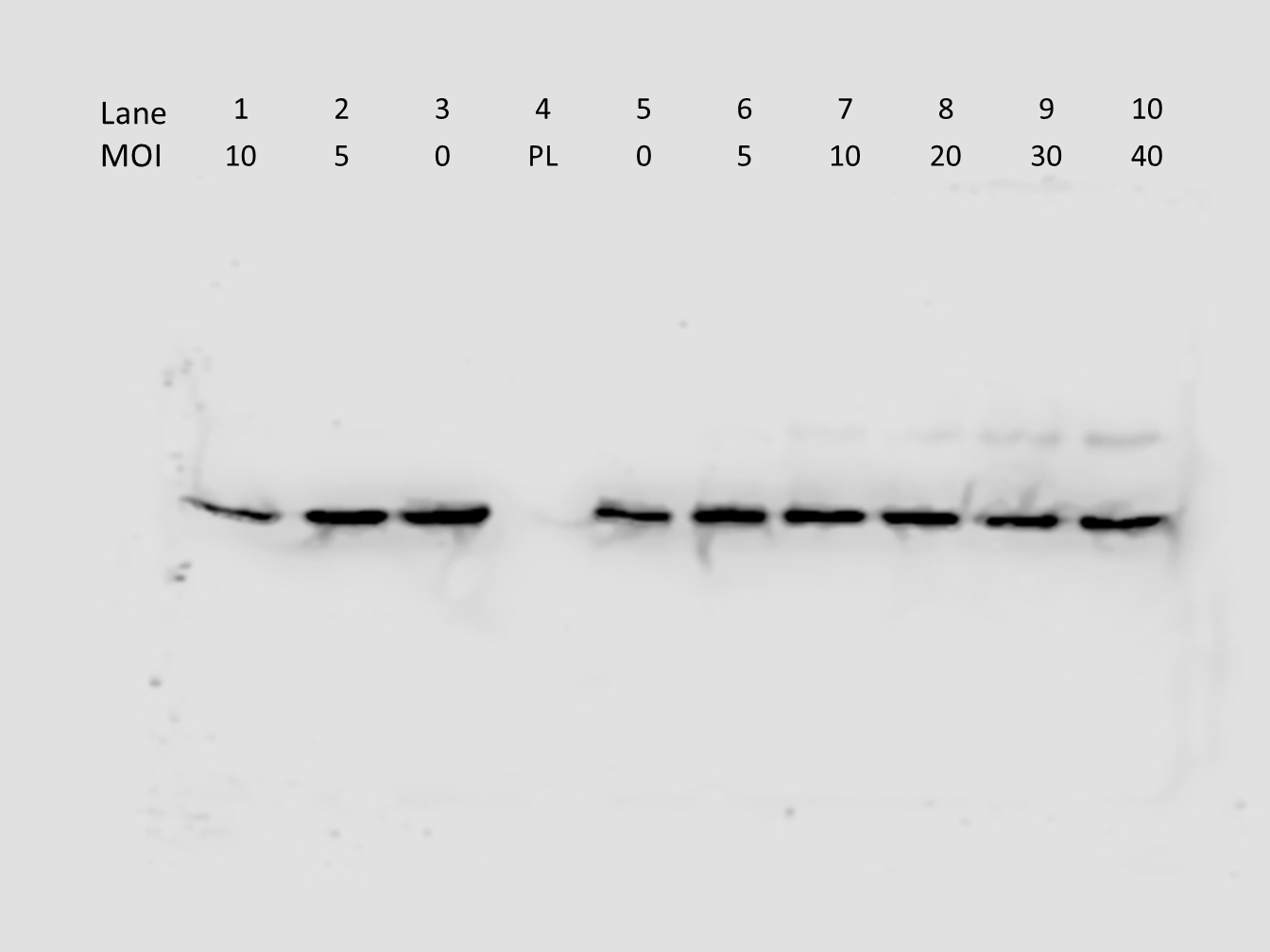

Supplement: Supplementary file 1 [file vaccines-09-00149-s001.zip › Supplementary/Original Figure 1 ACTIN.tif]

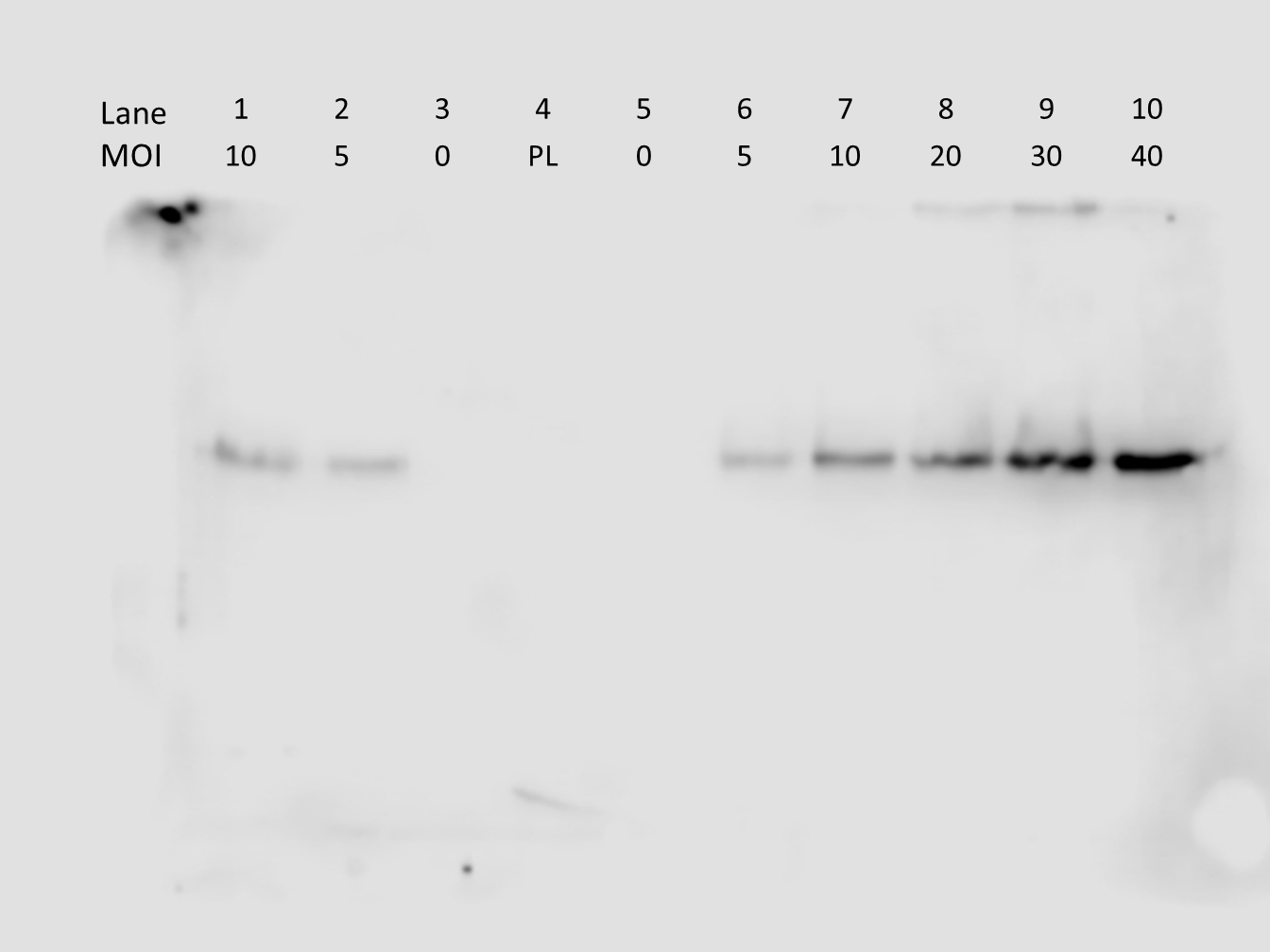

Supplement: Supplementary file 1 [file vaccines-09-00149-s001.zip › Supplementary/Original Figure 1 SP-SA-E7-4-1BBL.tif]
